# Suicide and Violence against Women in Azerbaijan: Risk Factors and Barriers for Seeking Mental Healthcare

**DOI:** 10.1192/j.eurpsy.2023.1180

**Published:** 2023-07-19

**Authors:** D. Alonzo, P. Zubaroglu

**Affiliations:** Fordham University, West Harrison, United States

## Abstract

**Introduction:**

Azerbaijan ranks among the top 3 countries with the highest rates of suicide in the Muslim world. Yet, research indicates an underestimate of risk due to cultural stigma related to suicidal behavior that may influence reporting and given that many Muslim-majority countries, where populations exceed 100 million, do not report any data on suicide rates. Violence against women also occurs at alarming rates in Azerbaijan and is a significant risk factor for suicide.

**Objectives:**

We examine perspectives towards suicide and violence against women and barriers to care among key stakeholders.

**Methods:**

Thirty qualitative interviews and 4 semi-structured focus groups were held with female survivors of suicide and mental health professionals working with individuals at risk of suicide to assess for perspectives on suicide and violence against women, factors influencing help-seeking, and the nature of existing resources.

**Results:**

Most participants viewed suicide (83%) and violence against women (73%) as problems. Nevertheless, 33% reported negative stereotypes regarding suicide and 50% reported psychological treatment as unaccepted in Azerbaijan. Findings highlight that domestic violence is the strongly identified as risk factor for suicide among women in Azerbaijan. Stigma and related cultural values regarding gender norms are significant contributors to violence against women and suicide. Existing services are underrecognized and perceived of as unavailable or insufficient.

**Conclusions:**

Employing a social determinants of health lens, multi-level programming is needed that spans micro (individual level supports), mezzo (family level supports), and macro (advocacy and outreach) levels to support a comprehensive strategy that beings with prevention and extends to address intervention, management, and capacity building to halt the increasing rates of suicide and deter violence against women.

**Disclosure of Interest:**

None Declared

